# Nearly Reversible Expansion and Shrinkage of Casein Microparticles Triggered by Extreme pH Changes

**DOI:** 10.3390/mi14030678

**Published:** 2023-03-19

**Authors:** Ronald Gebhardt, Thomas Pütz, Jann Schulte

**Affiliations:** Chair of Soft Matter Process Engineering (AVT.SMP), RWTH Aachen University, 52062 Aachen, Germany

**Keywords:** casein microparticle, swelling, systems dynamic modelling, colloidal calcium phosphate

## Abstract

Solvent flows in the fL/s range across the total surface of a casein microparticle cause its expansion and shrinkage. Microparticles prepared from the milk protein casein have a porous and flexible inner structure with water-filled channels and cavities. Solvent uptake occurs in two phases and results in disintegration if de-swelling is not triggered by acidification. So far, nothing is known about the reversibility of the swelling/de-swelling steps. We performed pH jump experiments between pH 11 and pH 1 on a single micro-particle and analyzed the swelling-induced size changes with system dynamics modeling. Both the swelling steps and the subsequent de-swelling process proceed reversibly and at an unchanged rate over a sequence of at least three pH exchange cycles. We observed that the duration of the first swelling step increased during the sequence, while the second step became shorter. Both of the time intervals are negatively correlated, while a statistical evaluation of only one swelling cycle for an ensemble of microparticles with different stabilities did not reveal any significant correlation between the two parameters. Our results indicate that the pH-induced swelling/shrinkage of casein microparticles is, to a large extent, reversible and only slightly influenced by the acid-induced decomposition of colloidal calcium phosphate.

## 1. Introduction

The volume changes caused by the swelling of hydrogels are of interest for a number of applications, such as sensors, actuators, soft-bodied robots, or drug delivery vehicles [1,2,3]. In sensors, for example, the change in volume is converted into an electrical output signal by suitable transducers [4]. Microgels are being developed, for example, as pH-responsive carriers for drug delivery through the gastrointestinal tract [5]. In general, microgels can be produced using microfluidic devices, emulsification techniques, photolithography, or micromolding [6]. We prepare proteinous microgels from the main protein component in milk using a structure preserving process, which we describe below. This milk protein casein is structurally different from most other proteins, due to their flowable and disordered structure [7]. Caseins are of interest for applications because they are, as an alternative to petrochemically produced polymers, easy to isolate, biodegradable, and biocompatible.

Under natural conditions, caseins exist as association colloids. These so-called casein micelles can be considered complex biological machines, due to their transport and storage properties for minerals, organic molecules, and proteins, as well as their high water-binding capacity [8]. Casein micelles are composed of a total of four different caseins (α_s1_-, α_s2_-, β-, and κ-casein) and colloidal calcium phosphate [9]. Their spherical shape with radii in the range of 50–200 nm can be ellipsoidly deformed by shear and extensional forces during filtration or drying [10,11]. The phosphoseryl clusters present in three of the four caseins (α_s1_-, α_s2_-, and β-caseins) bind colloidal calcium phosphate, which, together with divalent cations, cross-links casein chains [12]. They are also held together by hydrophobic and polar interactions, as well as hydrogen bonds [9,13]. Casein micelles can be described as a sponge-like structure interspersed with water-filled channels and cavities [14,15,16]. Colloidal stability is ensured by an outer κ-casein surface layer. Due to the good gelling properties and the ability to bind both hydrophobic substances and hydrophilic macromolecules, casein is a promising candidate to produce encapsulation matrices [17]. Applications as carriers for bioactive substances, such as curcumin and vitamins in food, antibacterial agents in animal feed, or drugs in cancer therapy have been described [18,19,20].

To preserve the structural and functional properties of casein micelles, we prepare casein microparticles by the depletion flocculation of casein micelles via volume exclusion of added pectin under neutral pH-conditions [21]. The resulting several µm-sized aggregates are then solidified into microparticles in the pectin matrix during film drying. Casein microparticles have sizes between 5–20 µm and swell in a basic environment in a two-step process before disintegrating at the end of the second swelling step [22,23]. The particle area can be observed microscopically and increases almost linearly with time during both swelling steps, with swelling step 2 always proceeding at a larger rate than swelling step 1 [23]. The expansion process can be suppressed or increased by increasing the free calcium concentration in the medium or by removing colloidal calcium phosphate by pre-treatment with the calcium chelator citrate [24,25]. A temperature post-treatment or an enzymatic cross-linking with the enzyme transglutaminase stabilizes the microparticles, so that they swell to an equilibrium final value, instead of disintegrating [26,27].

The microparticles retain their spherical shape throughout the entire swelling process, so that the two-step swelling kinetics can be simulated via two volume flows using systems dynamic modelling [22]. This modelling approach can be used to investigate the dynamic complexity for microscopic processes that arise from the influence of key variables or feedback from the system [28,29]. Our model predicts that expansion of the microscopically visible fine structure occurs during the first swelling step and that the volume increase and subsequent decay observed in the second swelling step is due to dissociation of the contacts between the fine structure elements [24]. The system feedback is caused by the increasing porosity and accessible hydratable volume during the swelling of the microparticles [23].

In contrast, the influence of key variables, such as pH or ionic environment, on the swelling flows is strongly influenced by the behavior of the casein micelles that make up the microparticles. During acidification, there is continuous solubilization of colloidal calcium phosphate inside the casein micelles [30]. The released hydrogen phosphate and the phosphoseryl residues on the casein are protonated, which leads to an increase in buffer capacity, with a maximum at pH 5.1. If the pH value drops further, the acidic amino acids of casein, such as aspartic and glutamic acid, are protonated, which also results in an increased buffer capacity at pH 3.5 [31]. During alkalinization, the free phosphate produced by the decomposition of colloidal calcium phosphate changes its degree of ionization, which increases the affinity for calcium and allows casein micelles to be demineralized [32,33]. As a result of the reduction of the mineral content in the aqueous phase, the solvent quality increases, which, together with the increase in electrostatic repulsion of the polymer chains, causes the disintegration of casein micelles at high pH values [34].

While the swelling of casein microparticles in a basic environment has been studied in detail, nothing is known about the possible de-swelling by acidification and the reversibility of the process. We, therefore, studied the expansion and shrinkage of a single microparticle over three cycles by alternating the exchange of the medium with aqueous HCL solution at pH 1 and NaOH solution at pH 11. How long the pH-dependent degrees of swelling are maintained is of particular interest, with regard to future applications, as microcapsules for cyclic and sustained release of bioactive substances.

## 2. Materials and Methods

### 2.1. Materials

The following working solutions were prepared for the individual preparation steps. A pectinase solution with an enzyme activity of 36 units/mL was prepared by adding 0.47 mL of pectinase from Aspergillus niger (Merck, Darmstadt, Germany) to 10 g of SMUF buffer for film hydrolysis. A casein dispersion (10% *w*/*w*) was prepared by adding 5 g casein powder to 45 g SMUF solution. The dispersion was stirred at room temperature for one hour, then at 4 °C for 4 h, and finally at 37 °C for another hour, in each case at 200 rpm. The pH of the casein dispersion was 6.7.

Casein microparticles were prepared by pectin-induced depletion flocculation of casein micelles and subsequent solidification of the resulting aggregates by film drying. Aggregates were initially formed by mixing a 0.3% micellar casein dispersion and a 3% pectin solution at pH 6.7 and room temperature. A total of 3.9 g of the aggregate solution was added to a glass petri dish and dried for 16 h under controlled laboratory conditions (T = 22 °C, RH 45%). The solidified casein micro-particles were then released from the film matrix by enzymatic hydrolysis with pectinase and separated from pectin residues by centrifugation at 1500 RCF for 10 min.

### 2.2. Swelling Experiments

**Figure 1:**
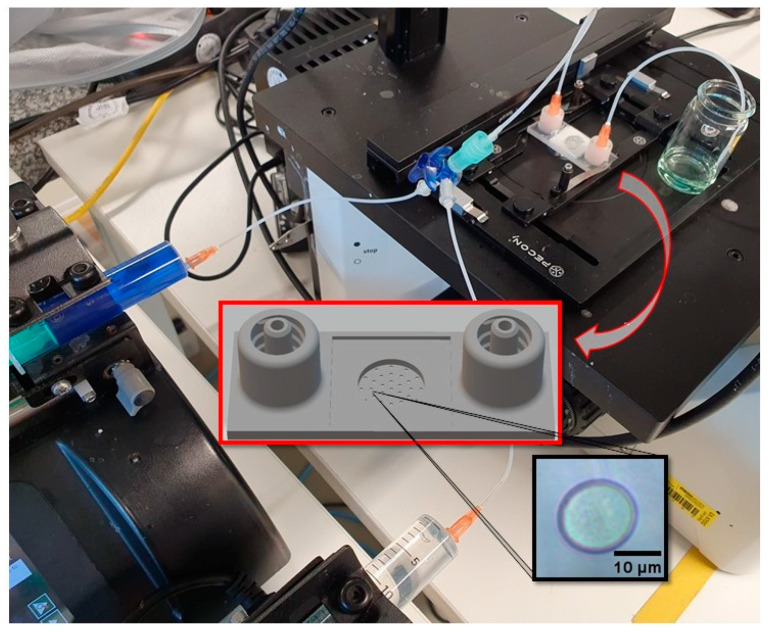
Experimental set-up consisting of inverted microscope with video monitoring, microfluidic swelling cell, syringe pumps for the repeated swelling cycle—experiments.

Repeated swelling tests on individual casein microparticles were carried out using the experimental setup shown in Figure 1, consisting of a swelling cell, two syringe pumps (PHD ULTRA™ Harvard Apparatus, Holliston, MA, USA) with adjustable flow rate of 0.05 mL per minute, inverted microscope, and detection unit. A microfluidic swelling cell (see Figure 1) with a sieve tray design was used as the sample environment. The circular sieve bottom has a diameter of 10 mm, and the cylindrical sieve holes inside have a diameter of 500 μm and a height of 1.6 mm. The construction plan of the swelling cell was designed with the CAD software Autodesk Inventor Professional 2018 and printed in one piece using the 3D printer Stratasys Objet Eden 260 V. The printing material VeroClear was heated up to 65 °C and printed with a spatial resolution of 16 μm. Afterwards, the printed microfluidic chip was sealed on the top and bottom with microscopic cover glasses. The swelling cell with sieve cell design allows for single particle investigations in different media. For this purpose, the sieve holes were filled individually at the beginning by sedimentation of the particles, and the swelling behavior was then observed with an inverted microscope (Leica DMIL LED, Leica Microsystems, GmbH, Wetzlar, Germany). For data acquisition, video was recorded at a rate of 2 frames per second using a Basler camera (Basler AG, Ahrensburg, Germany) installed on the microscope. Microscopic images were extracted from the video using the PyCharm script (version 2021.1.3, JetBrains, Prague, Czech Republic), and then ImageJ software (NIH, Bethesda, MD, USA) was used to calculate the area of the imaged particles.

The swelling and de-swelling of the microparticles was carried out by replacing the medium in the sieve holes with aqueous NaOH and HCl solution, which was alternately pumped through the swelling cell by syringe pumps at a flow rate of 50 µL/s via the valve shown in Figure 1. The sequence of swelling cycles was started by first replacing the buffer of the microparticles by an aqueous NaOH solution with a pH of 11. After doubling the observed particle area, the medium in the swelling cell was replaced by aqueous HCL solution with pH 1, and the de-swelling process was started. The pH change was monitored by detecting the color change of the indicator thymol blue. Two further swelling cycles were then carried out in the same way by the alternate exchange of basic and acidic solutions.

### 2.3. Dynamic Swelling Model and Data Analysis

To simulate the expansion and shrinkage kinetics of the casein microparticle, an extended dynamic swelling model was used, which is explained under Results and Discussion. For this purpose, a de-swelling step modelled by a volume outflow that depends on the current swelling volume and a rate coefficient were added to the swelling model of Schulte et al. [22]. The Euler integration method was used to solve the system of the underlying differential equations with a simulation step time of 0.25 s. The spherical approximation was applied to calculate the particle areas of the microparticle from the simulated volumes.

## 3. Results and Discussion

**Figure 2:**
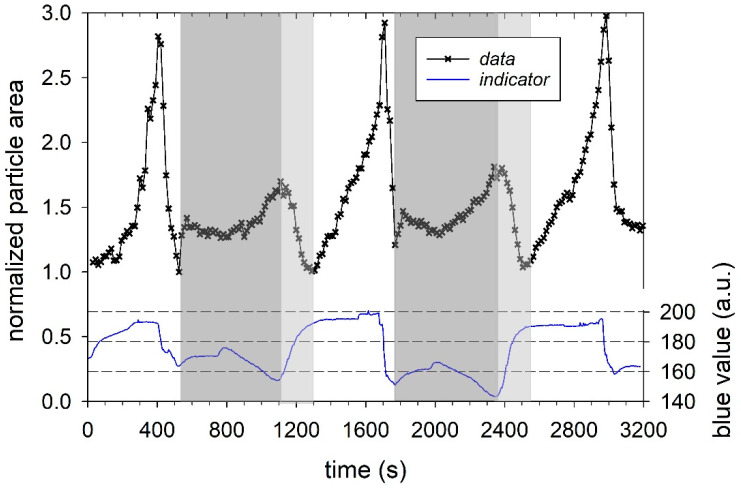
Swelling/de-swelling cycles of a single casein microparticle induced by pH-jumps. The blue value of the indicator shown below indicates the repeated pH progression between pH 11 and pH 1 and back again. Gray areas indicate the time between cycles, which were not considered for further analysis.

Previous experiments have shown that casein microparticles swell within a few minutes after replacing the buffer with swelling medium at pH 11, whereas they show little change in size in neutral and acidic medium [22]. We investigated the reversibility of the swelling process in detail by performing three consecutive swelling/de-swelling cycles by pH jump between pH 11 and pH 1 and back using the setup shown in Figure 1. The particle size and color of the added indicator changed, due to switching the aqueous media with different pH. Figure 2 shows the variation of the normalized particle area and indicator blue value as a function of time. The indicator signal was a distorted square pulse function with arbitrary intensity values of about 160 for pH 6.8 (at the beginning), 200 for pH 11, and about 150 for pH 1.

At the beginning of the three cycles, the particle area increased slowly and then more rapidly, in agreement with previous studies, due to a sequential swelling process [22]. However, at cycle 2 and 3, there is an initial decrease in particle area, despite an increase in pH (see light grey areas in Figure 2)—an observation we will discuss below. Using the first cycle as an example, the indicator signal between t = 400 and 500 s further shows that after the change from basic swelling medium at pH 11 to pH 1, the blue value first drops to a local minimum. As a result, the particle area is also reduced and, thus, the degree of swelling of the casein microparticles, as well. Similar correlated changes between pH and particle area also occur for the 2nd and 3rd swelling cycles after t = 1700 and 3000 s, respectively. The de-swelling of the microparticles is a consequence of increased cohesion between the caseins. This occurs because the net negative charge and, thus, the repulsion forces between the caseins are reduced at the beginning of the pH decrease. Furthermore, from pH 5.2 and below, there is also dissolution of colloidal calcium phosphate [13,35]. However, the stabilizing effect of colloidal calcium phosphate is compensated by other attractive interactions within the casein micelles after their disappearance [36,37]. At each cycle, the de-swelling process was initiated in such a way that no swelling-induced decomposition of casein microparticles, as observed in previous studies, occurred. For comparability of the individual cycles, the pH jumps were carried out with aqueous HCl solution in such a way that the maximum particle area during swelling was about 2.8 times the size at the beginning.

In addition, there are grayed out areas in Figure 2, which were not included in the analysis. In these areas, the pH value decrease weakened slightly at first, despite constant media exchange with aqueous HCL solution, before decreasing again more strongly. This, in turn, led first to a slight and later to a stronger swelling of the microparticle in the gray marked areas. The delayed decrease in pH can be attributed to the increased buffer capacity, which shows a maximum for casein micelles at pH 5.1. The effect has been attributed to the solubilization of colloidal calcium phosphate. After solubilization, protons bind to casein via protonation of the phosphoseryl residues and released phosphate [30,38]. The dissolution of colloidal calcium phosphate and the resulting residual charge on the phosphoseryl-residues could explain the transient increase in particle area to a plateau value in the grey regions between t = 600–800 s and 1800–2000 s. A little later, the particle area increases again, although the pH now decreases. Finally, at t = 1100 and t = 2300 s, the pH drops to its minimum value, and the normalized particle area reaches local maximum values of approx. 1.5. The reason for the renewed swelling is that, at this point, the pH has already fallen below the isoelectric point of the caseins (approx. pH 4.2). Amino groups are now positively charged, which, in turn, leads to increased electrostatic repulsion between the caseins, water binding, and swelling.

If the aqueous HCl solution is now replaced by NaOH solution with pH 11 during the next swelling cycle, the pH value rises again. Until the isoelectric point of the caseins is reached (areas marked in light grey), the microparticle shrinks back to its initial size, due to decreasing repulsion forces and de-swelling.

**Figure 3:**
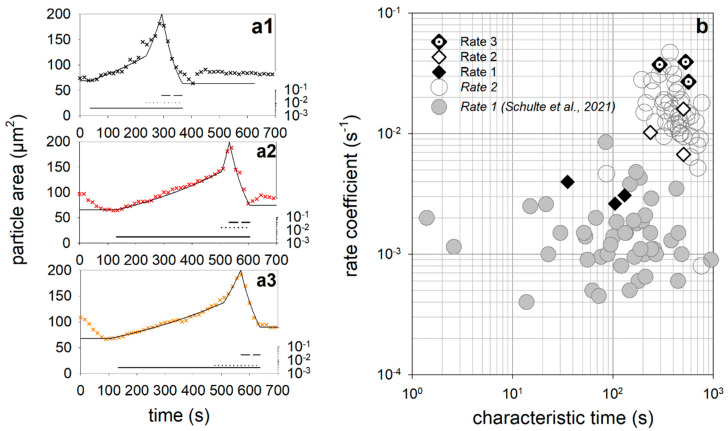
(**a1**−**a3**): Individual analysis of the three swelling peaks from Figure 2 with the extended sequential swelling model. (**b**) Plot of the simulated values for rate coefficient and characteristic time of the three process steps, in comparison with data from previous measurements [23].

**Figure 4:**
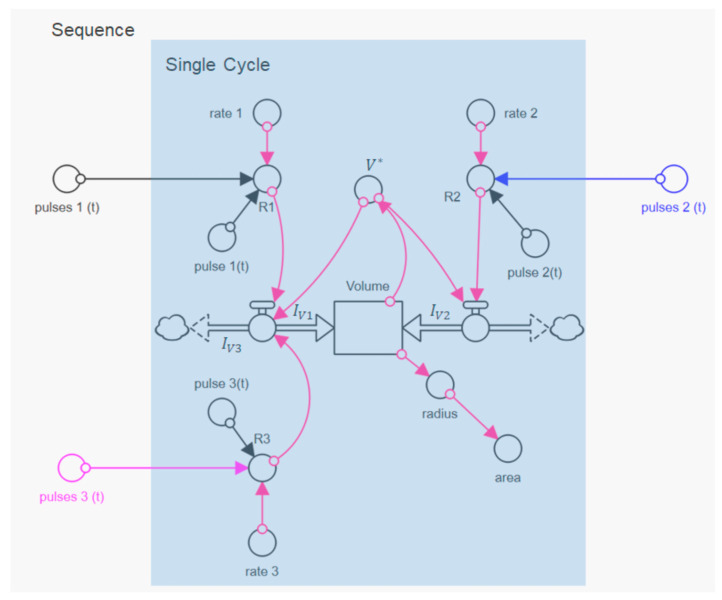
Dynamic STELLA model for the simulation of individual swelling and de-swelling cycles and a sequence of cycles.

We first analyzed the three swelling peaks in Figure 2 individually, using the data in the unmarked time intervals in Figure 2. In these time intervals, the change of the pH value was first with aqueous NaOH solution at pH 11 and then with aqueous HCL solution at pH 1, as shown by the distorted square pulses of the corresponding indicator signal. All three swelling cycles in Figure 3a1–a3 show a two-step swelling process, with the second swelling step proceeding faster than the first. This observation is consistent with previous swelling experiments on casein microparticles [22,23]. Studies with the calcium chelator citrate have shown that calcium influences the first swelling step, while no such dependence exists for the second swelling step [25]. After reaching the maximum swelling value, an even faster de-swelling takes place in all cases, as a result of the pH jump by aqueous HCl solution. We simulated the individual swelling experiments with a systems dynamic model, which is shown in Figure 4. This is a sequential swelling model type, according to Schulte et al. [22], which was extended for these special experiments by a third de-swelling step and pulse sequence functions.

For this purpose, we implemented another volume flow *I_V_*_3_ in the model, which negatively affects the rate of change of the particle volume via

(1) $$\dot{V}=I_{V1}+I_{V2}-I_{V3}$$

This allowed us to simulate the de-swelling process back to the initial state of the microparticle after switching to the acidic medium. Here, we used rectangular functions to simulate the individual swelling steps (*i* = 1…3) of the swelling cycle, whereas step functions were used in previous analyses of swelling curves that ended with the decay of casein microparticles [22]. The square pulses had the value 1 during the duration of the respective steps and 0 otherwise and were the sum of two step functions with characteristic times for start (*time_B_*) and end (*time_E_*).

(2) $${pulse}_{i}\left(t\right)=step\left(t-{time}_{B,i}\right)-step\left(t-{time}_{E}\right)$$

Multiplied by the rate coefficient of the respective step of the swelling cycle and the current volume of the microparticle, V* they define, at a given time, the volume flows *I_V_* in Equation (1), according to:

(3) $$I_{Vi}={pulse}_{i}\cdot {rate}_{i}\cdot V^{*}$$

The model simulations described the data during swelling and de-swelling with good accuracy, as shown by the solid lines. However, there are systematic deviations at the beginning and end of the swelling peaks, which can be attributed to the buffering effect after the solubilization of colloidal calcium phosphate, as well as to swelling effects below the isoelectric point of the caseins. The characteristic times and rate coefficients obtained from the individual simulations for the three process steps are shown in Figure 3b for comparison with corresponding values from earlier studies. For the first two swelling steps, corresponding comparative values exist, while the de-swelling step has not been investigated so far. The simulated rate coefficients for both swelling steps of all three cycles fall into the clusters of the corresponding values of previous measurements [23]. The values of the rate coefficients for the de-swelling step, studied here for the first time, are in the upper range of the cluster for the second swelling step. The absolute values, which are significantly larger than those for the two swelling steps are, however, strongly biased by the time intervals given by the pulse functions and must be viewed with caution. Since, in the simulation shown here, the swelling processes are also still active during the de-swelling step, the values of rate 3 might be too high. However, it can be noted that the rate coefficients of the de-swelling steps for all three cycles show similar values. Since all three rates of the swelling/de-swelling cycle do not change significantly during the sequence, a largely reversible association–dissociation mechanism is present. The comparatively large rates of the de-swelling step further indicate rapid structure recovery. Both indicate that the casein microparticles behave like a reversible polymer network, until they reach a state just before swelling-induced decay.

As suggested by the variability of the individual analyses in Figure 3b, all three swelling cycles of the sequence can be described by one set of rates, shown in Table 1.

**Figure 5:**
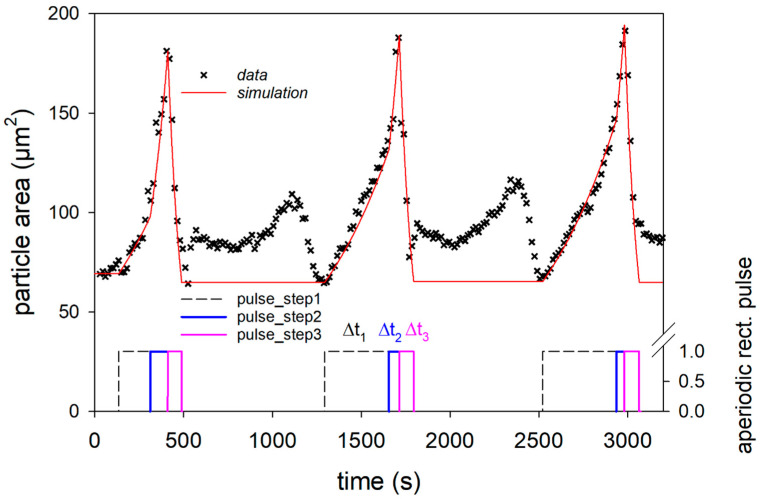
Simulation of the sequence of three swelling cycles using a sequential swelling model and aperiodic square pulses for each step.

For the simulation of the sequence of the three swelling/de-swelling cycles, only the characteristic times of the individual swelling steps had to be adapted (see Figure 5, below).

For this purpose, an aperiodic square pulse function, *pulses*(*t*), was used for all three swelling steps, which consists of the sum of the square pulses for the cycles *j* = 1.3:

(4) $$pulses\left(t\right)=\sum_{j=1}^{3}step\left(t-{time}_{A,j}\right)-step\left(t-{time}_{E,j}\right)$$

**Figure 6:**
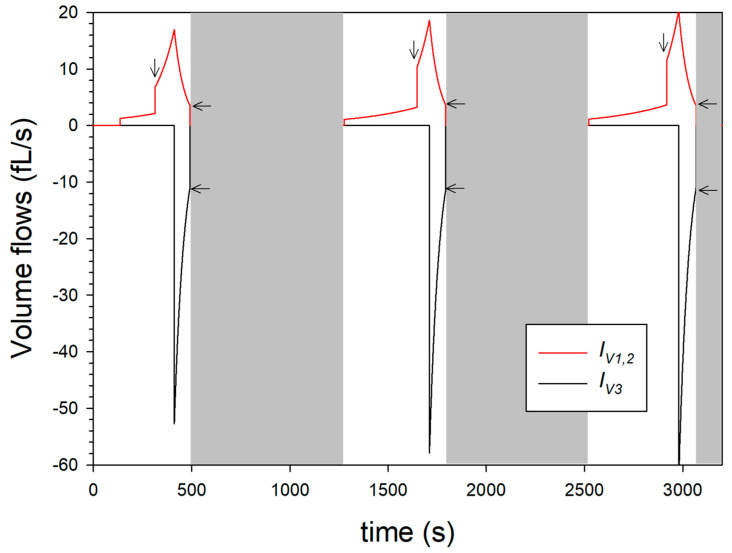
Simulated volume flows according to the systems dynamic model shown in Figure 4, which can be assigned to the swelling (*I_V_*_1,2_) or the de-swelling (*I_V_*_3_) of the microparticle.

The underlying model balance requires that size changes of the microparticle occur exclusively through the volume flows in the volume reservoir in Figure 4. In contrast, sources or sinks for volumes within the microparticle were not considered. This approach seems reasonable because caseins have an inherently unfolded and flexible structure [7], so that volume changes, due to refolding, can be excluded. Furthermore, it can be assumed that the internal structure of microparticles, as known for casein micelles, has solvent-filled channels and cavities [15], which do not cause any volume change upon structural reorientation. However, contributions resulting from water binding to ionized groups, which would lead to a volume reduction, due to the electrostrictive effect [39,40], have not yet been taken into account. Figure 6 shows the time course of the volume flows that can be assigned to the expansion or shrinkage process of the microparticle, according to the model approach.

While the volume flows *I_V_*_1,2_ together reach maximum values of 15 to 20 pL/s during alkalinization, the volume outflow starting after acidification is significantly shorter and reaches maximum values of −50 to −60 fL/s. The jump in volume inflow (see vertical arrows) is particularly drastic and caused by the beginning of swelling step 2. When the maximum volume flow *I_V_*_1,2_ is reached, the pH drop inside the microparticle causes a de-swelling, which is modelled via the volume flow *I_V_*_3_. Due to the maximum volume expansion (V*), *I_V_*_3_ initially has maximum absolute values of approx. 50 fL/s and more according to the dynamic model in Figure 4 and then drops, similar to the swelling flow *I_V_*_1,2_, due to the shrinkage. At the beginning of the grey areas, the inflows and outflows (indicated by horizontal arrows) are not equal in absolute value, so that the microparticle has not reached a state of equilibrium at these times. The plot of the volume flow in Figure 6 also shows that, from cycle to cycle, swelling step 1 takes longer, while swelling step 2 is shortened, and de-swelling step 3 remains unchanged.

**Figure 7:**
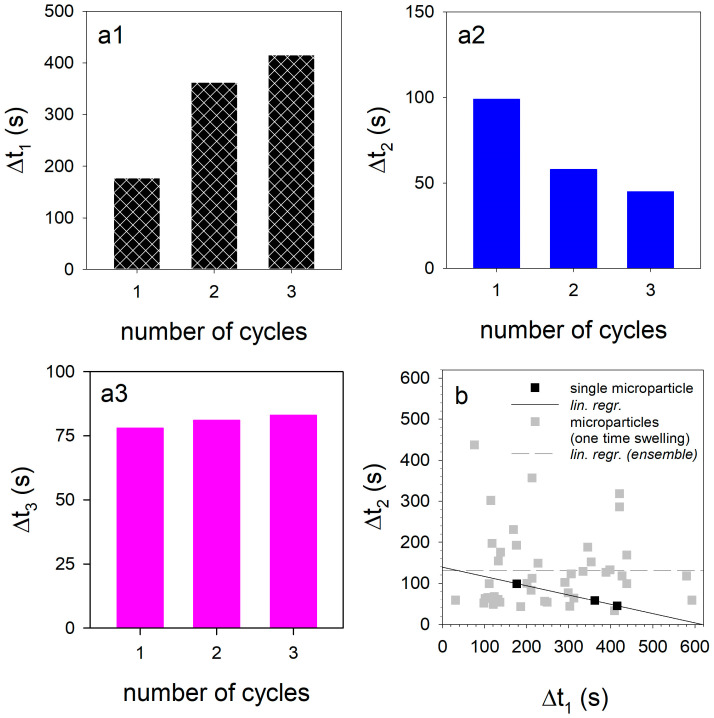
(**a1**−**a3**): Variation of the pulse durations of the individual swelling steps from cycle to cycle. (**b**) Scatter plot demonstrating the relationship between the durations of the two swelling steps.

Figure 7a1–a3 shows how the duration of the swelling/de-swelling steps changes from cycle to cycle. The duration of the first step increases from 180 s (first cycle) to 360 s (second cycle) to 400 s for cycle 3. In contrast, the time of the second swelling step decreases from 100 s via 60 s to 45 s, while the duration of the third de-swelling step remains almost unchanged at approx. 75 s. The time intervals of the first two swelling steps for the three cycles are almost perfectly negatively correlated (black dots and solid line in Figure 7b). A short first swelling step causes a longer second swelling step and vice versa. The swelling of the microparticle would theoretically take only about 140 s without swelling step 1, but 620 s without swelling step 2, as Figure 7b indicates. In contrast, no such correlation could be found for casein microparticles when only one swelling cycle was performed. We have already investigated the influence of different drying conditions on swelling and the subsequent decomposition on 32 individual microparticles at pH11 in a statistical analysis [23].

Due to different internal structuring and initial hydration levels, changes in the swelling kinetics resulted, so that correlations between the parameters of the swelling model could be specifically investigated. For casein microparticles with higher initial water content, both swelling steps started delayed (positive correlation between the characteristic times). The characteristic times of both swelling steps were delayed the more, the smaller the rate of the second swelling step was (negative correlations in each case). We re-evaluated the data set from Schulte et al. [23] by also determining the time intervals ∆t1 and ∆t2 and checked them for a linear correlation. In the scatter plot in Figure 7b, the corresponding values representing the duration of the two swelling steps of each of the 32 casein microparticles are also shown as grey squares. Based on the point cloud, no correlation could be found between the two time intervals (grey dashed line). It also follows, from the dataset from Schulte et al. [23], that swelling step 1 dominates the total duration of the swelling process (∆t1 + ∆t2) as soon as ∆t1 > 120 s. Swelling step 2 takes an average of 130 s, regardless of how much the duration of swelling step 1 is delayed due to structural changes.

However, when considering a sequence over three cycles for the single casein microparticle, the duration of the second swelling cycle decreases linearly with the duration of the first step. We attribute this effect to the stepwise degradation of colloidal calcium phosphate, which exists dispersed in the micellar building blocks of the microparticle. During the change to swelling medium at pH 11, the colloidal calcium form initially remains unaffected [32]. However, after subsequent acidification, the colloidal calcium phosphate is gradually dissolved, while the concentration of free calcium and phosphate increases [33,41]. Previous swelling studies have shown that only swelling step 1 is influenced by calcium chelator citrate, whereas no calcium phosphate bonds are involved in swelling step 2, which concerns the contacts between the µm-sized building blocks of the casein microparticles [25]. The nature of the casein–casein contacts does not change dramatically during the sequence, as the rates of the swelling steps remain unchanged from cycle to cycle (see the simulation in Figure 5 with parameter values in Table 1). Instead, changes in the duration of the swelling steps occur during the sequence. We assume that this is due to a change in the number of interaction contacts triggered by calcium phosphate in various forms. Colloidal calcium phosphate interlocks individual casein chains [9,16] in the beginning and during the first swelling step. However, if a de-swelling step follows afterwards, the colloidal calcium phosphate decomposes due to acidification. As a result, the polymer chains can move closer together, creating a larger interaction surface between the caseins. The dissociation of the additional casein–casein contacts and the hydration of the released contact areas requires more and more time for swelling step 1 during cycle 2 and 3 and leads to the increase of ∆t1 in Figure 7a1.

The negative correlation between the duration of the first and the second swelling step could be due to the effect of the phosphate released from the dissociating colloidal calcium phosphate. At alkaline pH, the ionization state of this phosphate changes from HPO4^2−^ to PO4^3−^, which has a greater affinity for calcium and, for example, removes calcium bound via phosphoseryl groups to caseins [32,33]. Because of the formation of poorly soluble calcium phosphate, the mineral content in the aqueous phase is reduced, which improves the solvent quality for caseins within the microparticle [34]. As a result of the reduced cohesive interactions and increased repulsion, there is less contact area between the microstructure within the microparticle, and the time of the second swelling step ∆t2 is reduced accordingly. From cycle to cycle, the colloidal calcium phosphate decomposes more and more during de-swelling in the acidic environment. The duration of swelling step 1 in the 3rd cycle now increases to a lesser extent, since the area of interaction between the caseins grows less. The same applies to the decrease in the length of swelling step 2. Because less PO4^3−^ is available, smaller amounts of calcium are also removed from the contact surfaces of the microparticle building blocks by the formation of poorly soluble calcium phosphate. As a result, there is less increase in repulsion forces, and swelling step 2 shortens less than what was the case in swelling cycle 2. Another indication of the stepwise, and eventually complete, decomposition of colloidal calcium phosphate can be observed in Figure 2. While the microparticles at the beginning of the gray-marked areas after cycles 1 and 2 swell again due to the residual charges on the phosphoseryl-residues, this effect can no longer be observed during acidification in cycle 3. For the statistical investigation of the particle ensemble, however, only one swelling cycle with a pH jump from pH 6.8 to pH 11 was considered. Since no further pH jumps to pH 1 and to pH 11 were carried out, there was also no modulation of the cohesive forces between the caseins triggered by the decomposition of the colloidal calcium phosphate. Consequently, no correlation between the lengths of the swelling steps could be found.

## 4. Conclusions

Our results on a casein microparticles demonstrate that the pH-induced swelling/shrinking is highly reversible over three cycles. The entire sequence could be described with one rate for each swelling/de-swelling step, despite the change to extreme pH values. This suggests that, during the repetitive expansion/shrinkage, the stability-relevant structures within the microparticle are preserved. Due to the pH stability, microparticles could be used as a protective encapsulation matrix for enzymes at extreme pH values to perform, e.g., a coupled acidic and enzymatic hydrolysis. The casein microparticles could also be used as sensors for changing environmental conditions, such as pH or calcium ions, because of their repeatable, rapid swelling. For this purpose, they could either be adsorbed on electrodes and used as electrochemical biosensors [42], or the calibrated size changes could be observed microscopically. However, a delay of the first swelling step occurred during the swelling/de-swelling sequence with a perfect negative correlation to the duration of the second swelling step. It is known from the literature that solubilization of the casein-bound colloidal calcium phosphate occurs after acidification, leading to an increase of soluble phosphate and calcium concentration in the medium [30,38]. The reaction is accompanied by increased buffering at pH 5.1, resulting in a delayed phase of the indicator signal, which we observe during the de-swelling step. Our studies indicate that the solubilization of micellar calcium phosphate, which was linked to caseins via phosphoserine clusters, provides a strong correlation between the duration of the two swelling steps. After repeated buffer exchange to basic, the meanwhile free phoshoseryl residues inside the µm-sized building blocks can additionally bind water and delay swelling step 1. The high-affinity form of phosphate resulting from the decomposition of colloidal calcium phosphate removes the calcium bound to casein, which increases the charge density at the contact surfaces of the building blocks and shortens the second swelling step. The influence of the decomposition of colloidal calcium phosphate on the swelling mechanism could be investigated in the future using FLIM fluorescence microscopy studies on free calcium, which was recently detected with this technique after the acidification of micellar casein fibers [43]. In further studies, we will use an improved experimental setup to investigate the repeatability of the pH-induced size changes for a large number of casein microparticles over many swelling cycles. The aim is also to further optimize the pulse-like exchange of the medium, in terms of the pulse shape and periodicity. In the future, the in situ measurements of further milieu and process parameters should be carried out in an extended experimental set-up, in order to investigate the swelling behavior of casein microparticles in more detail.

## Figures and Tables

**Table 1:** Rates used to simulate of the sequence of swelling cycles shown in Figure 5.

Parameter	Value [s^−1^]
rate 1	0.00293
rate 2	0.00640
rate 3	0.02898

## Data Availability

Not applicable.
